# Supplementary material for: Unconventional Magnetism and Band Gap Formation in LiFePO4: Consequence of Polyanion Induced Non-planarity
Source: Sci Rep. 2016 Jan 21;6:19573. doi: 10.1038/srep19573 (PMC4726275; doi:10.1038/srep19573)
Supplement: Supplementary Information [file srep19573-s1.doc]

**Supplementary Information**

Unconventional Magnetism and Band Gap Formation in LiFePO_4_: Consequence of Polyanion Induced Non-planarity

Ajit Jena and B. R. K. Nanda

*Condensed Matter Theory and Computational Lab*

Department of Physics, Indian Institute of Technology Madras

Chennai, India, 600036

*e-mail address:* [*nandab@iitm.ac.in*](mailto:nandab@iitm.ac.in)

**S1.** Illustration of the unstable low-spin (non-magnetic) and stable high-spin state in (Li)FePO_4_.


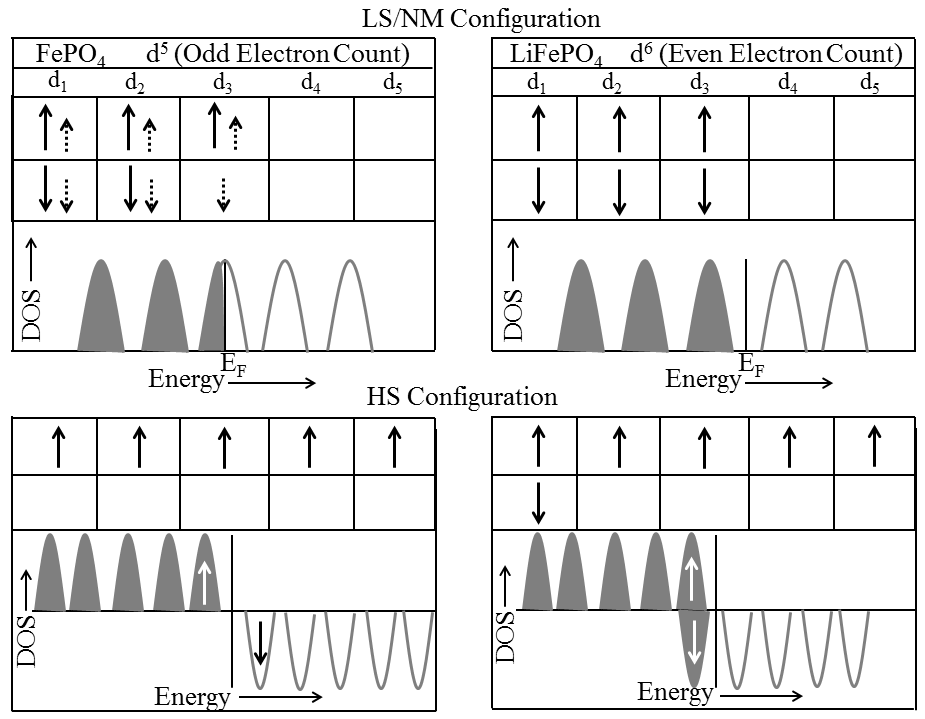


Fig. 1: Electronic configuration for the non-magnetic (or LS configuration) and HS configuration for FePO_4_ and LiFePO_4_. The dashed line represents the non-magnetic configuration for FePO_4_. HS configuration stabilizes the system due to Hund’s rule coupling.

For FePO_4_, the available unpaired electron does not exist in the hypothetical non-magnetic structure since the spin-degree of freedom is discounted in this case. As each state has the ability to occupy two electrons, the lower lying d-states are partially occupied to provide a metallic solution. In Fig. 1, the dashed lines schematically illustrate this aspect which is also obtained from the DFT – GGA band structure as shown in Fig. 2 (left). For even number of d electrons (LiFePO_4_) the LS configuration coincides with the non-magnetic structure. HS configuration stabilizes the system. At higher temperature, the spins are randomly oriented to provide a paramagnetic insulating phase. As the temperature is lowered the spins are ordered antiferromagnetically as discussed in the paper. Contrary to LiFePO_4_, DFT+GGA ground state of FePO_4_ has a wide band gap (~ 1.5 eV) as can be seen from Fig. 2.


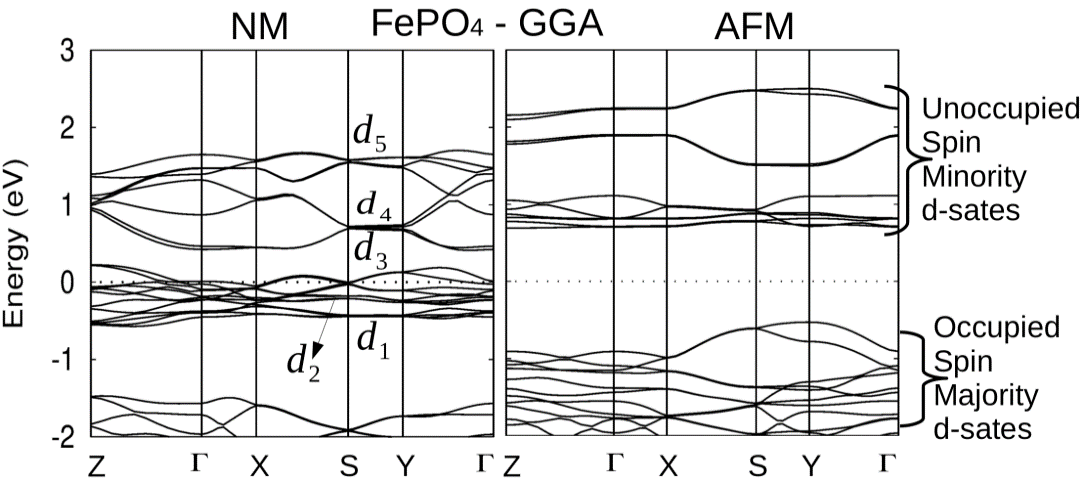


Fig. 2: FePO4 band structure in the nonmagnetic (NM) and ground state antiferromagnetic (AFM) configurations.

**S2.** Localized O-p states.


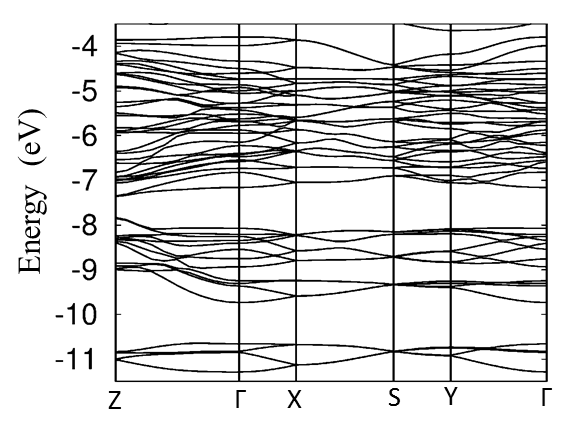


Fig. 3: The O-p bands for the ground state AFM configuration of LiFePO_4_. The bands are narrow with band width less than or close to 1 eV.

In most of the transition metal oxides (TMO) O-p states have larger band width. However, in the family of transition metal olivine phosphates, with negligible p-p and p-d covalent interaction the O-p bands are narrow like the d-states. This is due to the fact that the P and O form strong ionic bonds and behave like a single entity.

**S3.**  Antiferromagnetic and insulating (AFI) band structure of LiMPO_4_ (M = Cr, Mn, Co and Ni)

*
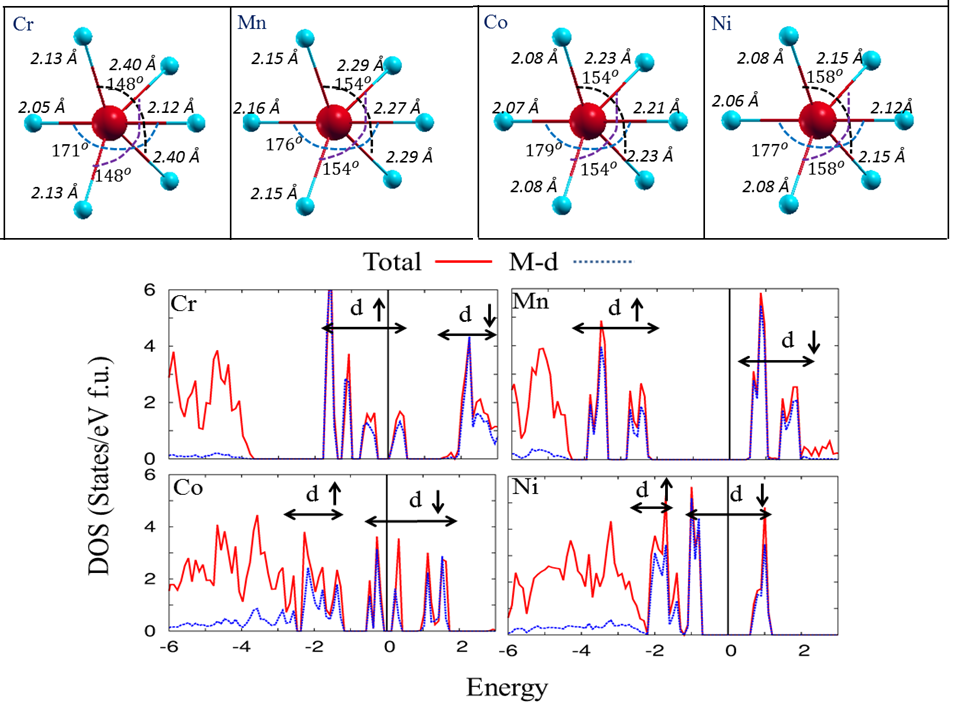
*

Fig.4: Upper panel: distorted MO_6_ complex in LiMPO_4_ (M = Cr, Mn, Co, Ni). Lower panel: The ground state AFM density of states of LiMPO_4_. Each of them shows insulating behaviour.

As discussed in the paper, the antiferromagnetic and insulating (AFI) behaviour in LiFePO_4_ is primarily attributed to the formation of cluster like states which are the outcome of the asymmetric crystal field. Following Fig. 4, the same analysis can be extended to the other members of LiMPO_4_.
